# Woody encroachment induced earlier and extended growing season in boreal wetland ecosystems

**DOI:** 10.3389/fpls.2024.1413896

**Published:** 2024-05-15

**Authors:** Hongchao Sun, Wen J. Wang, Zhihua Liu, Lei Wang, Suri G. Bao, Shengjie Ba, Yu Cong

**Affiliations:** ^1^ State Key Laboratory of Black Soils Conservation and Utilization, Northeast Institute of Geography and Agroecology, Chinese Academy of Sciences, Changchun, China; ^2^ University of Chinese Academy of Sciences, College of Resources and Environment, Beijing, China; ^3^ Institute of Applied Ecology, Chinese Academy of Sciences, Shenyang, China; ^4^ School of Geographical Sciences, Northeast Normal University, Changchun, China

**Keywords:** northern ecosystem, climate change, phenology, vegetation greenness, woody encroachment

## Abstract

Woody plant encroachment (WPE), a widespread ecological phenomenon globally, has significant impacts on ecosystem structure and functions. However, little is known about how WPE affects phenology in wetland ecosystems of middle and high latitudes. Here, we investigated the regional-scale effects of WPE on the start (SOS), peak (POS), end (EOS), and length (GSL) of the growing season in boreal wetland ecosystems, and their underlying mechanisms, using remote sensing dataset during 2001–2016. Our results showed that WPE advanced the annual SOS and POS, while delaying EOS and extending GSL in boreal wetlands with these impacts increasing over time. When boreal wetland ecosystems were fully encroached by woody plants, the SOS and POS were advanced by 12.17 and 5.65 days, respectively, the EOS was postponed by 2.74 days, and the GSL was extended by 15.21 days. We also found that the impacts of WPE on wetland SOS were predominantly attributed to the increased degree of WPE (α), while climatic factors played a more significant role in controlling the POS and EOS responses to WPE. Climate change not only directly influenced phenological responses of wetlands to WPE but also exerted indirect effects by regulating soil moisture and α. Winter precipitation and spring temperature primarily determined the effects of WPE on SOS, while its impacts on POS were mainly controlled by winter precipitation, summer temperature, and precipitation, and the effects on EOS were mainly determined by winter precipitation, summer temperature, and autumn temperature. Our findings offer new insights into the understanding of the interaction between WPE and wetland ecosystems, emphasizing the significance of considering WPE effects to ensure accurate assessments of phenology changes.

## 1 Introduction

Woody plant encroachment (WPE), a widespread ecological phenomenon globally, has been often attributed to changes in disturbance regimes (e.g., fire suppression, overgrazing) and climate change (e.g., rising temperature and atmospheric CO_2_ concentration) (Criado et al., 2020; Drees et al., 2023; Yang et al., 2023). Boreal wetland ecosystems in the Northern Hemisphere, particularly susceptible to climate change, have experienced notable impacts due to WPE (Armitage et al., 2021; Zhang et al., 2021). The resultant changes in wetland ecosystem phenology and productivity, along with shifts in community composition and structure induced by WPE, can significantly impact carbon and water cycles, soil nutrients and biodiversity (Daryanto et al., 2019; Zhao et al., 2023; Ding and Eldridge, 2024). However, there is still a significant gap in understanding how WPE affects ecosystem phenology in boreal wetlands.

Vegetation phenology reflects the growth and developmental rhythms shaped by the long-term adaptation of vegetation to environmental changes, representing a periodic natural variation process within a year (Caparros-Santiago et al., 2021). It serves not only as a sensitive indicator of responses to climate change but also plays a crucial role in the carbon-water cycle process between terrestrial ecosystems and the atmosphere (Imhoff et al., 2004; Bai et al., 2020). For example, the spring green-up (Start of Season, SOS) and late-season senescence (End of Season, EOS) directly determine the length of the growing season (GSL), thereby influencing the duration of photosynthesis in the canopy (Wu et al., 2012). Thus, accurately capturing the changes in different phenological stages helps reduce the uncertainty in estimating land model carbon and water fluxes.

Comprehending the mechanisms driving plant phenological shifts is crucial for forecasting future changes and their ecological consequences (Asam et al., 2018). Most studies suggest that the main driving mechanisms behind phenological changes are global temperature rise, elevated atmospheric CO_2_ concentration, nutrient deposition, and alterations in precipitation patterns (Cleland et al., 2006; Menzel et al., 2006; Prevéy and Seastedt, 2014). However, ecological processes such as WPE could concurrently exert great influences on the phenology through changing plant functional traits in ecosystems (Valencia et al., 2016). WPE generally leads to increases in leaf area, which is related to interception and absorption of solar radiation, resource allocation, water uptake, thus promoting earlier plants growth and longer growing season (Gross et al., 2007; Mitchard and Flintrop, 2013). In addition, the leaf morphology could change with WPE, due to the physiological differences between woody and wetland plants (Eldridge et al., 2011). Leaf morphology is an indicator of plant relative growth rate and utilization of resources (Wright et al., 2004). It serves as a descriptor of dominant plant strategies; generally, a higher leaf morphology is associated with a fast-growing strategy (Freschet et al., 2011). Moreover, deep-rooted woody plants can access deep soil water before snowmelt and spring rainfall, leading to an earlier onset of root activity (Sutinen et al., 2009), which in turn can promote the onset of spring surface phenology (Caldararu et al., 2012). The impacts of ecosystem phenology resulting from changes in dominant species may even surpass the effects of climate change (Xu et al., 2015; Zettlemoyer et al., 2019). For example, Wilsey et al. (2018) observed that phenological changes resulting from the transition from native to exotic species were much greater than those induced by temperature increases in experimental grasslands in the central U.S.

Climate change and WPE may also interact to have complex influences on vegetation growth and phenology in wetland ecosystems. Climate change can directly and indirectly shape the phenological responses of wetland ecosystems to WPE in middle to high latitudes (Piao et al., 2020; Mohl et al., 2022). Thus, a better understanding of how WPE impacts phenology metrics on boreal wetland ecosystems, as well as the underlying mechanisms linking climate change, is imperative for accurately predicting vegetation responses and feedbacks to climate change.

Since traditional experimental methods often have limited capacity to capture high spatial heterogeneity and long-term vegetation changes, accumulated large amounts of satellite data offer great opportunity to reveal changes in vegetation phenology and composition over continuous spatiotemporal scales. This study built upon our previous efforts in mapping tree cover fractions in boreal wetland ecosystems by integrating high spatial resolution (HSR) images, Landsat composites and machine learning regression modeling. We aimed to investigate regional effects of WPE on ecosystem phenology (SOS, POS, EOS, and GSL) in boreal wetlands of Northeast China, as well as their underlying mechanism, utilizing remote sensing datasets spanning from 2001 to 2016. Specifically, our study addressed three research questions: (1) What were the effects of WPE on ecosystem phenology in these middle-high latitude wetlands? (2) What were the annual dynamics of these effects? (3) What were the underlying mechanisms influencing the effects of WPE on wetland ecosystem phenology?

## 2 Materials and methods

### 2.1 Study area

**Figure 1:**
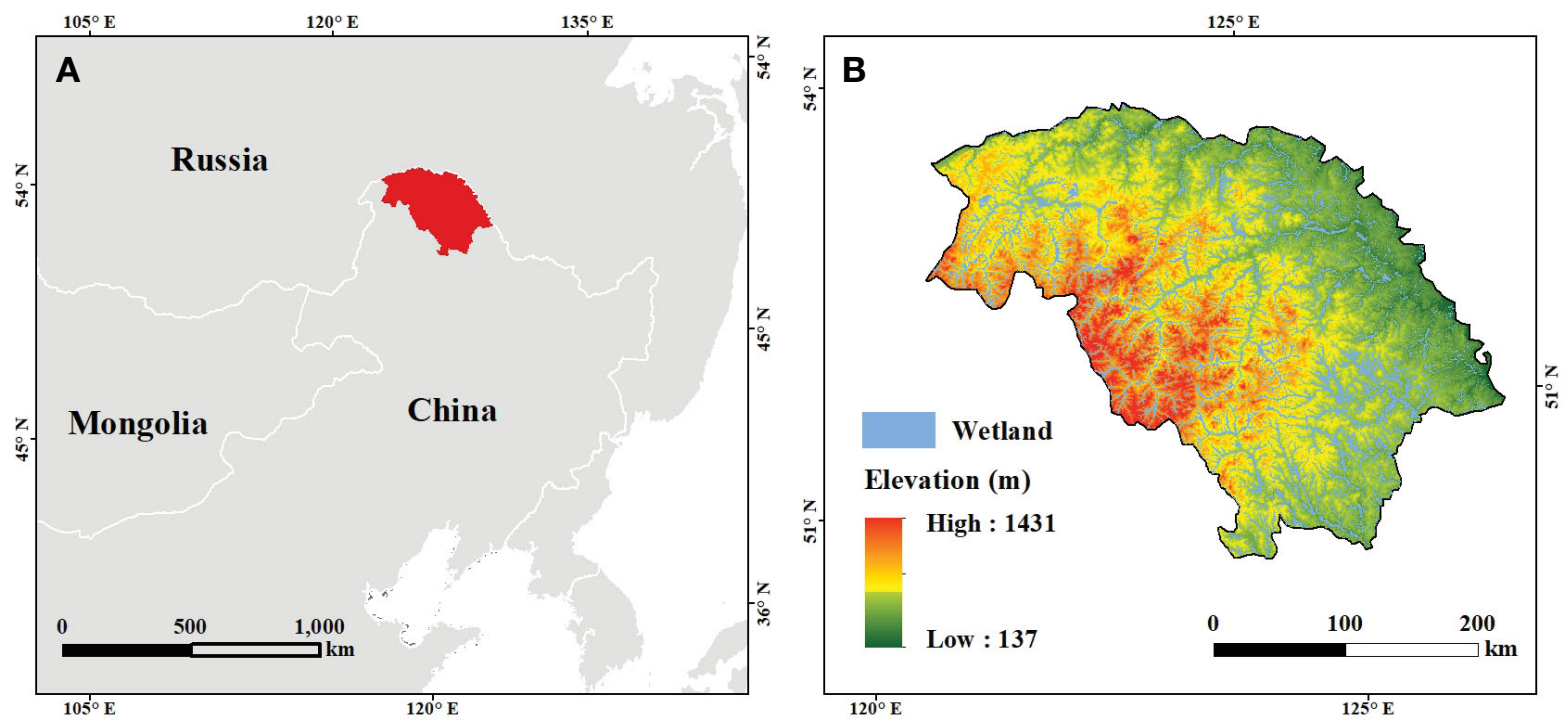
Location of the study area **(A)** and distribution of wetlands **(B)**.

Our study area was located on the northern slope of the Great Xing’an Mountains (121° 12´–127° 0´E and 50° 10´–53° 33´N) in Northeast China, encompassing an area of 8.46 × 10^4^ km^2^ (**Figure 1**). The area has a cool temperate monsoon climate, with long severe winters and short warm summers. The annual average temperature remains approximately -2.2°C, with an annual precipitation averaging about 500 mm (reference period: 2001 to 2016). The region is primarily covered by cool boreal coniferous forests, characterized by Dahurian larch (*Larix gmelini* (Rupr.) Kuzen) and white birch (*Betula platyphylla* Suk.) as the dominant trees. Boreal ecosystems in this region are less influenced by anthropogenic disturbances, because of small population. In recent years, with rising temperatures, WPE has becoming increasingly noticeable in widely distributed bush swamps and marshes in this region, which were the focus of this study (**Figure 1**).

### 2.2 Data

#### 2.2.1 Tree cover fraction maps in boreal wetland ecosystems

We devised a workflow for producing 30-m fractional coverage maps of trees in wetlands using high spatial resolution (HSR) imagery including WorldView-2 and SuperView-1 satellites, as well as Landsat time series data spanning 2001-2016. Initially, we employed supervised object-based classification of HSR images over selected sites across the study area. Subsequently, these classification maps served as training data to achieve wall-to-wall mapping of fractional tree covers using the Landsat data and Artificial Neural Networks (ANN). The accuracy assessment of the ANN model revealed a high performance for mapping tree cover fractions, with each map demonstrating an accuracy exceeding 90% (**Supplementary Figure S1**). To align with the timeline of MODIS datasets, we used tree maps from the two most recent periods (2001-2008 and 2009-2016). To investigate how the current climate conditions affect the effects of WPE on phenology, we also combined these two maps to generate a tree map for 2001–2016. We then aggregated these 30-m tree cover fraction maps to resolution of 500 m, which were used for subsequent analyses alongside 500-m MODIS data. All tree cover maps and following datasets were then extracted for our analyses using the wetland map of the entire study area in 2012 that was obtained from the National Earth System Science Data Center, National Science & Technology Infrastructure of China (http://www.geodata.cn).

#### 2.2.2 Land cover dynamics phenology products

We employed the MODIS Land Cover Dynamics phenology product (MCD12Q2, Collection 6) at a 500-m spatial resolution to identify the difference in phenology (SOS, POS and EOS) between wetlands with and without WPE from 2001 to 2016. Phenological metrics from MCD12Q2 were annually estimated by fitting a logistic function over the MODIS EVI time series (Descals et al., 2020). Additionally, we derived the GSL as the indicator of the duration between SOS and EOS.

#### 2.2.3 Climatic data

We acquired monthly mean temperature (Tmean) and total monthly precipitation (Pre) data at a spatial resolution of 1 km from the National Earth System Science Data Center (http://www.geodata.cn/) for the time period 2001-2016. The climatic dataset was downscaled from the Climatic Research Unit (CRU) time series data using the Delta downscaling method (Peng et al., 2019).

Correlation coefficients between each phenological metric and temperature, precipitation, within different lagged months before each phenological metric were calculated to identify the most related periods of climatic factors (Piao et al., 2006). The climatic factors from the two months preceding each phenological metric were ultimately identified as their primary drivers. The average temperature, precipitation, and soil moisture in April and May (spring) were identified as significant climatic factors influencing SOS; the climate conditions in June and July (summer) mainly impacted POS, while the climatic factors in September and October (autumn) controlled EOS. We also included significant impacts of autumn temperature of the previous year (September to October of last year) and winter precipitation (November to March of the subsequent year) to take into account of climate change legacy effects on vegetation growth in the Northern Hemisphere (Kelsey et al., 2021).

#### 2.2.4 Soil moisture

We acquired monthly.1° soil moisture (SM) data for four soil depths (i.e., 0–7, 7–28, 28–100 and 100–289 cm; hereafter, SM1-4) from the Climate Data Store (CDS) infrastructure (https://cds.climate.copernicus.eu) for the time period 2001-2016. The SM data was the fifth generation of global climate reanalysis data (ERA5) provided by the European Centre for Medium-Range Weather Forecasts (ECMWF). It has been widely used for the evaluation of SM products from other sources and the ecohydrological model (Zohaib and Choi, 2020). We aggregated the SM data for four soil depths by depth-weighted averaging (i.e. averaged by weighting values for each depth interval).

### 2.3 Methods

#### 2.3.1 Window searching strategy

We used a window searching strategy to enable a comparison between tree-encroached wetland and pure wetland pixels within each window (Wang et al., 2021). This approach ensured that pixels in a given window shared a similar environment, thereby attributing the variance between the two pixels to varying degrees of encroachment rather than other factors (**Supplementary Figure S2**). We selected the samples of tree-encroached wetlands pixels with relatively significant tree cover (at least 10%) and used the adjacent pure wetland pixels to construct pair samples.

Since a small window size would not provide adequate number of pure wetland pixels for analysis and a large window may be influenced by spatial heterogeneity, we thus utilized two window sizes, 11 by 11 pixels (~5 by 5 km) and 21 by 21 (~10 by 10 km) pixels at 500 m spatial resolution. Finally, for two window sizes, we obtained 1311 and 1456 samples for the time period of 2001-2008, and 1103 and 1149 samples for the time period 2009-2016, respectively. Next, we assessed regional effects of WPE on SOS, POS, EOS and GSL in boreal wetlands using these window samples.

#### 2.3.2 The effects of WPE on phenology in boreal wetland ecosystems

We firstly compared each phenological metric (SOS, POS, EOS and GSL) between the selected encroached and pure wetland samples at the regional scale to reveal the annual effects of WPE on ecosystem phenology in boreal wetlands. We then calculated the multi-year average relative effects of varying degrees (α) of WPE on wetland phenology (*β*_SOS_, *β*_POS_, *β*_EOS_ and *β*_GSL_) as the difference between encroached and pure wetland samples in each sampled window (Equations 1). These parameters represent localized effects within specific windows. Given the limited sample size with 100% WPE, we employed a methodology from previous studies by Wang et al. (2018), Wang et al. (2021) to quantify the specific effects of 100% WPE (full encroachment) on wetland phenology (*θ*_SOS_, *θ*_POS_, *θ*_EOS_ and *θ*_GSL_) at the regional scale (Equations 2, **Appendix Text. S1**).


(1) $$\beta_{\text{SOS}}={\text{SOS}}_{\text{TEW}}-{\text{SOS}}_{\text{wetland}}$$

(2) $$\theta_{\text{SOS}}={\text{SOS}}_{\text{tree}}-{\text{ SOS}}_{\text{wetland}}$$

We assessed the multi-year average of *θ*_SOS_, *θ*_POS_, *θ*_EOS_ and *θ*_GSL_ for two periods of 2001–2008 and 2009–2016. Additionally, we assessed the annual *θ*_SOS_, *θ*_POS_, *θ*_EOS_ and *θ*_GSL_ spanning the entire period 2001–2016 to unveil the interannual dynamics of WPE effects.

#### 2.3.3 Drivers of responses of phenology to WPE

Since structural equation modeling (SEM) captures the direct, indirect, and overall impacts among variables, it serves as a valuable tool in ecological and climatological studies (Shi et al., 2016; Fauchald et al., 2017). To disentangle effects of drivers, including degree of WPE (α), climate and soil moisture in determining the effects of WPE on wetland phenology (SOS, POS and EOS), we constructed two piecewise SEMs using “piecewiseSEM” package in R. Based on empirical knowledge, we formulated a foundational structural model and then fine-tuned it based on observed model fits. We evaluated the fit of each model using a χ2-test of Fisher’s C statistic, ensuring that it fell below the significance level (P > 0.05). The data utilized for the analysis with SEMs consisted of 932 sample windows covering the period from 2001 to 2016, as described in the Methods section (2.3.1). We also conducted sensitivity analysis to elucidate the distinct responses of phenology (SOS, POS, and EOS) to climate change in encroached and pure wetlands based on sample windows.

## 3 Results

### 3.1 WPE effects on phenology in boreal wetlands

**Figure 2:**
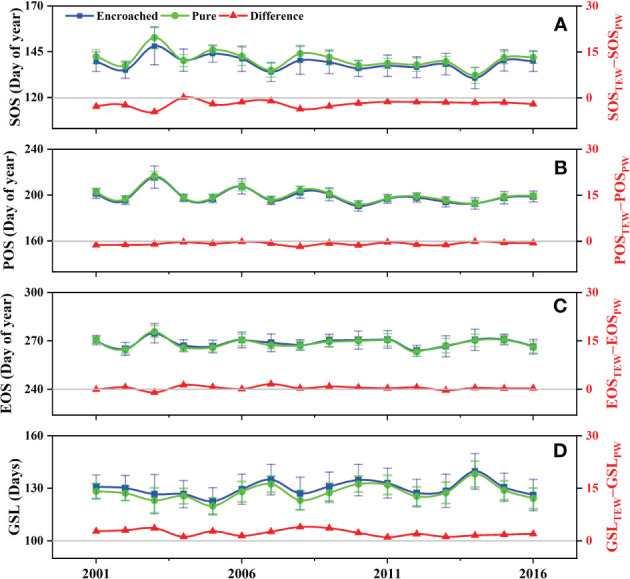
Comparisons of the **(A)** start (SOS), **(B)** peak (POS), **(C)** end (EOS), and **(D)** length (GSL) of growing season between pure wetlands and tree-encroached wetlands.

From 2001 to 2016, the regional onset of SOS and POS of tree-encroached wetlands generally preceded that of pure wetlands, indicating that WPE promoted the advancement of the greening-up and peak-growing periods in boreal wetland ecosystems. EOS and GSL typically occurred later and lasted longer than those of pure wetlands, indicating that WPE delayed the senescence period and prolonged the overall growing season (**Figure 2**). On average, WPE resulted in a significant advancement of SOS and POS by 1.97 days and 0.85 days (p< 0.01) in boreal wetland ecosystems, respectively. EOS was delayed by 0.36 days (p< 0.05), and GSL was extended by 2.25 days (p< 0.01).

**Figure 3:**
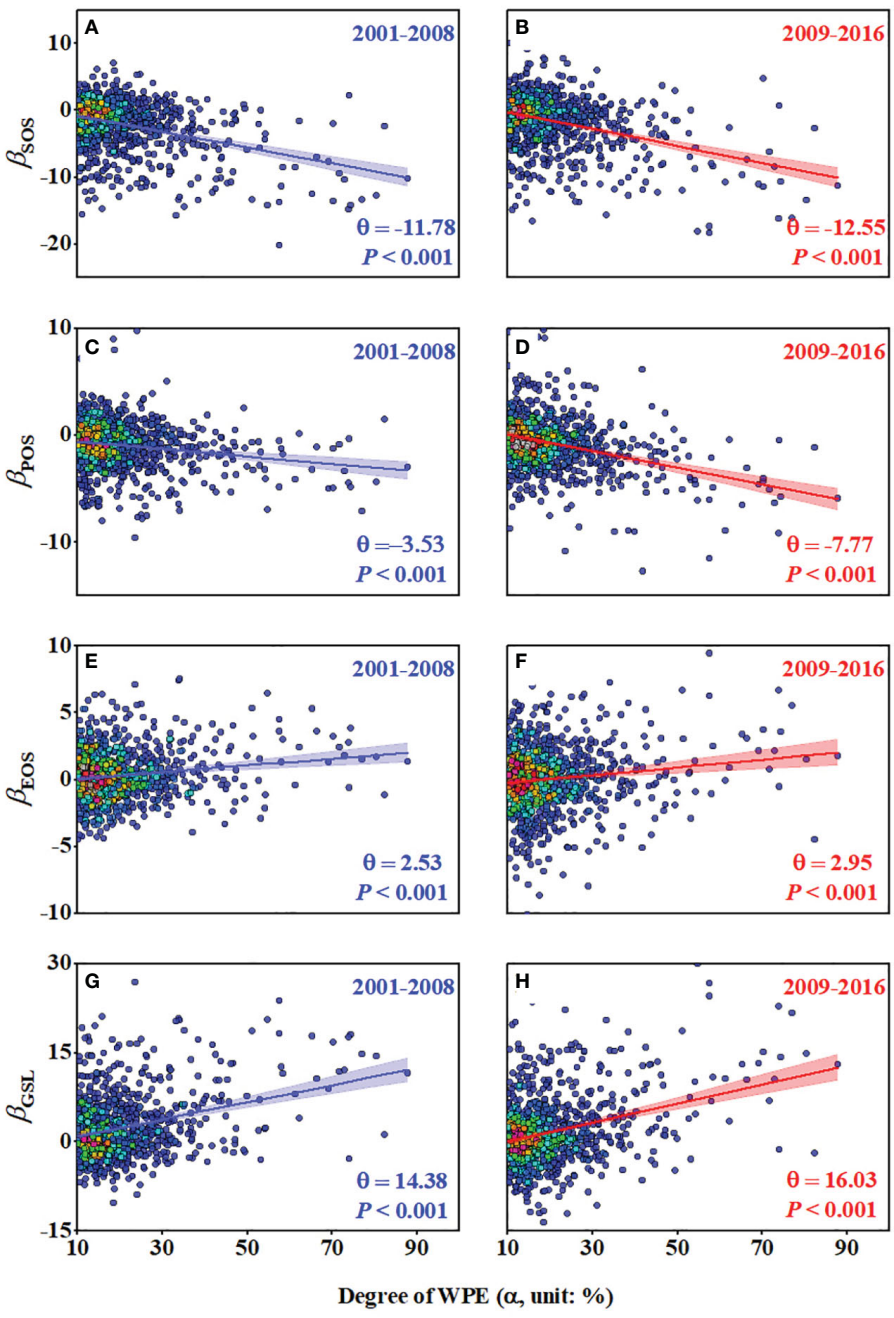
The multi-year average effects of varying degrees (α) of WPE (*β*_SOS_, *β*_POS_, *β*_EOS_ and *β*_GSL_) and the specific effects of 100% WPE (*θ*_SOS_, *θ*_POS_, *θ*_EOS_ and *θ*_GSL_) on wetland SOS **(A, B)**, POS **(C, D)**, EOS **(E, F)** and GSL **(G, H)** based on the selected neighborhood windows of 11 × 11 pixels.

The effects of WPE on wetland SOS, POS, EOS and GSL (*β*_SOS_, *β*_POS_, *β*_EOS_ and *β*_GSL_) statistically increased with the degree of WPE (α) during two periods 2001-2008 and 2009-2016 (**Figure 3**). *θ*_SOS_ was -11.78 days and -12.55 days, *θ*_POS_ was -3.53 days and -7.77 days, *θ*_EOS_ was -2.53 days and -2.95 days and *θ*_GSL_ was 14.38 days and 16.03 days in 2001-2008 and 2009-2016, respectively. We obtained similar results using the neighborhood window of 21 × 21 pixels (**Supplementary Figure S3**).

**Figure 4:**
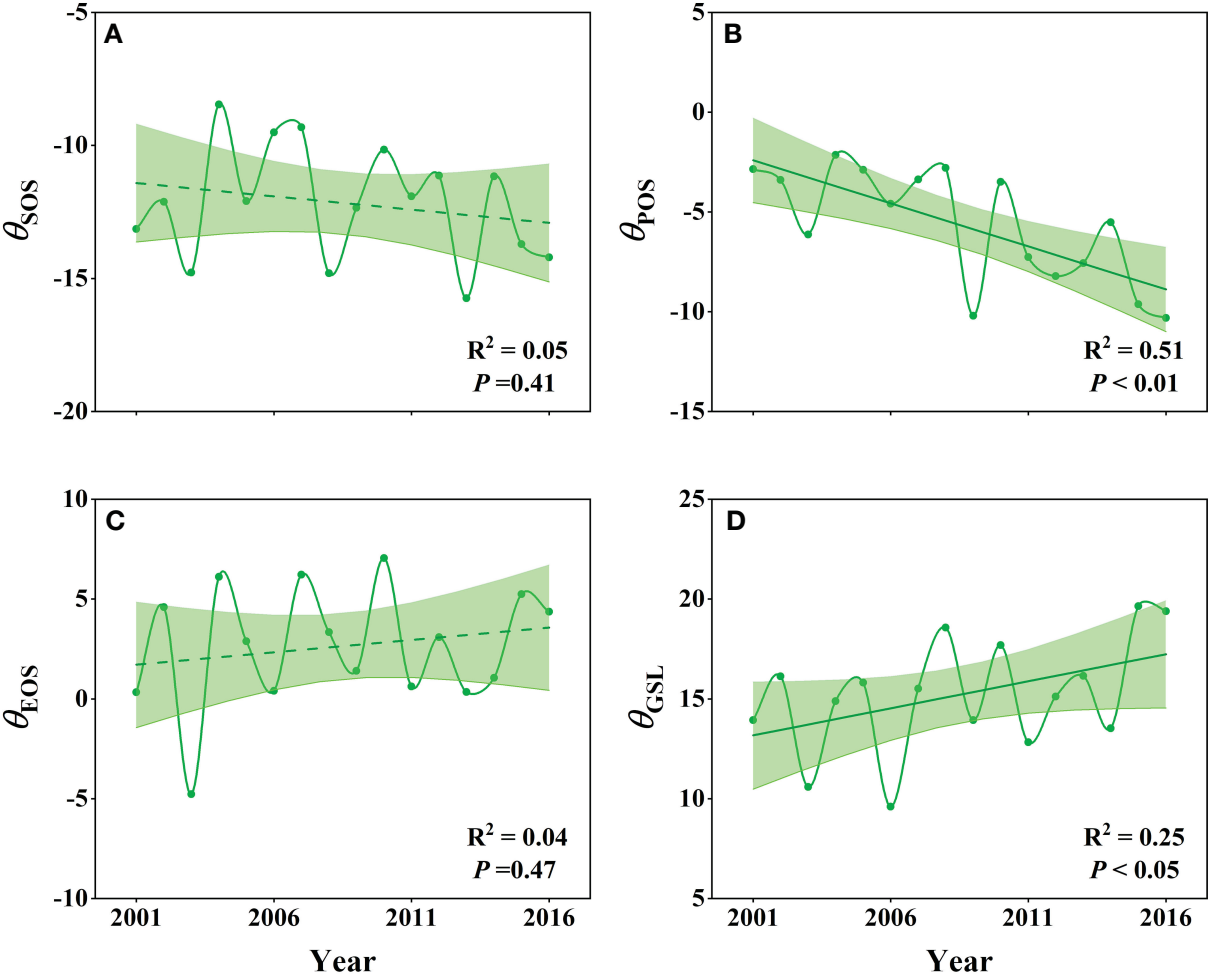
Interannual dynamics **(A–D)** of the specific effects of 100% WPE on wetland SOS (*θ*_SOS_), POS (*θ*_POS_), EOS (*θ*_EOS_) and GSL (*θ*_GSL_) based on the selected neighborhood windows of 11 × 11 pixels.

Our analysis of the *θ*_SOS_, *θ*_POS_, *θ*_EOS_ and *θ*_GSL_ responses revealed distinct interannual patterns (**Figure 4**). The *θ*_SOS_ showed a slightly advanced trend, while *θ*_POS_ exhibited a significantly advanced trend from 2001 to 2016 (P<0.01), with *θ*_SOS_ ranging from -15.74 to -8.46, and *θ*_POS_ ranging from -10.31 to -2.14 (**Figures 4A, B**). Additionally, *θ*_EOS_ showed a slightly delayed trend, and *θ*_GSL_ demonstrated a significantly extended trend (P< 0.05), with *θ*_EOS_ ranging from -4.78 to 7.05, and *θ*_GSl_ ranging from 9.6 to 19.64 (**Figures 4C, D**). We obtained similar results using the neighborhood window of 21 × 21 pixels (**Supplementary Figure S4**).

### 3.2 The driving factors of WPE effects on phenology in boreal wetlands

**Figure 5:**
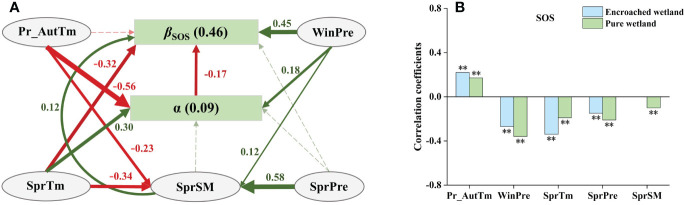
**(A)** SEM analyses revealed the impacts of climatic drivers on the response of wetland SOS to WPE (Fisher’s C=0.44, P=0.80). Green solid arrows represented positive paths, yellow solid arrows represented negative paths, and dashed arrows indicated nonsignificant paths. Arrow width indicated the strength of the relationship. The values adjacent to arrows were standardized path coefficients (P< 0.05). Numbers in brackets indicated the R^2^ of the response variables explained by their drivers. **(B)** Climatic sensitivity differences of SOS between encroached and pure wetlands (Significance level: ** p < 0.01).

The optimal SEM of *β*_SOS_ (**Figure 5A**) revealed the total effects of the degree of WPE (α), climatic factors and SM were −0.17, 0.12 and 0.12, respectively, which indicated that α had the greatest impact on *β*_SOS._ Winter precipitation (WinPre) had the strongest positive direct effects on *β*_SOS_, and also indirectly influenced it positively by controlling the degree of WPE (α) and spring soil moisture (SprSM). The spring mean temperature (SprTM) showed strongest negative direct effects and bidirectional direct effects on *β*_SOS_. In contrast, autumn temperature of the previous year (Pr_AutTm) indirectly influenced *β*_SOS_ negatively by regulating α, while spring precipitation (SprPre) indirectly affected *β*_SOS_ by controlling SprSM. Sensitivity analysis indicated that the SOS of pure wetlands was more correlated with WinPre, SprPre, and SprSM, while the SOS of encroached wetlands was more sensitive to Pr_AutTm and SprTM (**Figure 5B**).

**Figure 6:**
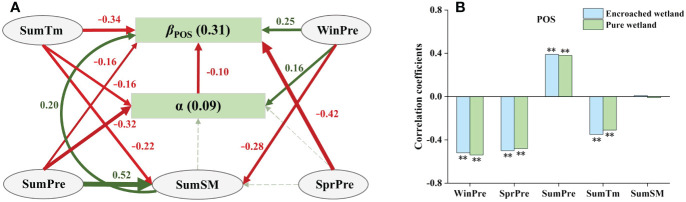
**(A)** SEM analyses revealed the impacts of climatic drivers on the response of wetland POS to WPE (Fisher’s C=2.54, P=0.28). **(B)** Climatic sensitivity differences of POS between encroached and pure wetlands (Significance level: ** p < 0.01).

The total effects of degree of WPE (α), climatic factors and SM were −0.10, −0.70 and 0.20, respectively, indicating that climatic factors had the greatest relative importance in influencing *β*_POS._ Summer mean temperature (SumTm) not only directly suppressed *β*_POS_, but also indirectly inhibited it by regulating α and summer soil moisture (SumSM). Summer precipitation (SumPre) and SprPre had negative direct effects on *β*_POS_, while SumPre also had indirect effects on it by increasing SumSM and inhibiting α. Winpre not only had strongest positive direct effect on *β*_POS_, but also had indirect effects on it by affecting SumSM and α (**Figure 6A**). Sensitivity analysis indicated minimal difference in the correlation between POS and climatic factors in encroached and pure wetlands (**Figure 6B**).

**Figure 7:**
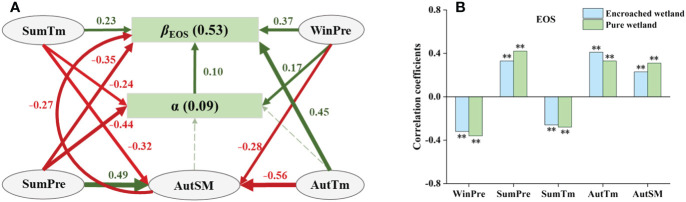
**(A)** SEM analyses revealed the impacts of climatic drivers on the response of wetland EOS to WPE (Fisher’s C=1.30, P=0.52). **(B)** Climatic sensitivity differences of EOS between encroached and pure wetlands (Significance level: ** p < 0.01).

The total effects of degree of WPE (α), climatic factors and SM were 0.10, 0.57 and −0.27, respectively, which indicated that climatic factors had the greatest impact on *β*_EOS._ Autumn mean temperature (AutTm), SumTM and WinPre had positive direct effects on *β*_EOS_ (**Figure 7A**), while SumPre showed negative direct effects on it. These four climatic factors also had indirect effects on *β*_EOS_ by mediating autumn soil moisture (AutSM) and α. Sensitivity analysis revealed that EOS in pure wetlands was more responsive to WinPre, SumPre, SumTm, and AutSM, while EOS in encroached wetlands was more sensitive to AutTm (**Figure 7B**).

## 4 Discussion

### 4.1 Effects of WPE on phenology in boreal wetlands

Our research showed significant phenological impacts of WPE on boreal wetland ecosystems. WPE advanced green-up onset and peak, delayed senescence, and extended growing season of wetlands. While studies on phenological effects of WPE were limited, a recent study near our study area found that compared to wetland plants, woody plants such as forests and shrubs typically had earlier spring green-up and later senescence, resulting in a longer growing season (Fu et al., 2022), which were consistent with our results. Above- and below-ground phenology exhibit asymmetrical timing, as evidenced by root growth preceding leaf growth by several weeks in Arctic shrub-graminoid communities (Radville et al., 2016). The deep roots of woody plants can utilize deep soil water before snowmelt and spring rainfall, allowing them to initiate root phenology earlier (Lambais et al., 2017; Zheng et al., 2022), consequently promoting the onset of green-up above grounds (Piao et al., 2019). Additionally, larger leaf area of woody plants enables the encroached woody wetland ecosystems to receive more solar radiation, further promoting the onset of spring phenology (Tooley et al., 2022; Li et al., 2023). Previous studies have found a significant correlation between the onset of green-up and peak greenness, with earlier green-up often indicating an earlier peak (Gonsamo et al., 2018). This could be attributed to inherent regulatory mechanisms in plants, such as programmed cell death and leaf lifespan (Reich et al., 1992; Buermann et al., 2018). Therefore, the advancement of green-up due to woody encroachment was accompanied by an earlier peak greenness. WPE also led to later senescence dates, prolonging the growing season thus the photosynthetically active period, with significant implications for regional carbon and water cycling.

Our results indicated that when boreal wetland ecosystems were completely encroached by woody plants, SOS and POS were advanced by 12.17 days and 5.65 days, respectively, while EOS was delayed by 2.74 days, and GSL extended by 15.21 days. Previous phenological research in China indicated a strong correlation between the green-up date and the average temperature of the preceding two to three months (Ma and Zhou, 2012; Wu et al., 2016). A 1°C rise in spring temperatures could advance the green-up date by approximately 7.5 days, while the similar temperature increase in autumn might delay the senescence date by about 3.8 days (Piao et al., 2006). Based on our results, phenological differences between encroached and pure wetlands exceeded phenology changes typically anticipated from climate change alone, particularly regarding leaf green-up. This finding aligned with a previous study on the phenological effects of plant invasion into grasslands in the central United States (Wilsey et al., 2018). They found that grasslands dominated by exotic species greened on average 10.7 days earlier and senesced 36 days later compared to those dominated by native species. The difference in senescence between native and exotic species was significantly greater than that attributable to temperature increases. The phenological changes induced by WPE may disrupt synchrony between species and trophic resources (Kharouba et al., 2018) and modify the competitive dynamics among coexisting species (Zhu et al., 2018), thereby having significant implications for biodiversity and ecosystem functioning (Woods et al., 2022). Therefore, gaining a deeper understanding of the mechanisms behind WPE-induced phenological changes can inform restoration strategies for native wetland vegetation and enhancing wetland management (Han et al., 2022; Helfter et al., 2022).

It’s important to note that we observed a gradual increase in the influence of woody expansion on surface phenology over time, consistent with findings from previous studies suggesting that the effect of woody expansion on carbon sequestration becomes apparent only after a certain age (Kelleway et al., 2017). This is associated with the relationship between the annual growth pattern of woody plants and tree age. Compared to older trees, younger trees tend to grow faster, but the date of maximum growth rate for older trees precedes that of younger trees (Jiang et al., 2021). The survey results of stand age showed 80% of encroached trees in our study area were under 30 years old (**Supplementary Figure S5**), suggesting that the effects of WPE might not yet have peaked. Hence, ongoing monitoring of how WPE affects the phenology in boreal wetlands remains crucial.

### 4.2 Contributions of drivers to the effects of WPE

Our study revealed that the influence of WPE on wetland phenology was modulated by climatic factors. Previous autumn warming delays senescence, potentially leading to a later entry into endodormancy, which may shorten the chilling accumulation period and increase the thermal demand for green-up onset, possibly resulting in a later SOS (Asse et al., 2018; Chen et al., 2019). Since woody plants have later dormancy dates, SOS in encroached wetlands was later with previous autumn warming compared to pure wetlands (**Figure 6B**). The magnitude of this negative difference in SOS between encroached and pure wetlands decreased statistically. Therefore, autumn temperatures showed a positive relationship with the effects of WPE on SOS. In the Northern Hemisphere, winter precipitation, primarily snow, insulates soil, boosts microbial activity, and supplies water for early plant growth (Zheng et al., 2022). Consequently, increased winter precipitation significantly advanced the onset of vegetation green-up, showing a negative correlation with SOS in both pure and encroached wetlands. Since shallow-rooted wetland plants are more sensitive to winter precipitation, winter precipitation has a more pronounced effect on advancing the green-up period in pure wetlands, reducing the negative difference in SOS between pure and encroached wetlands. Spring warming shortens the heating accumulation and stimulates the growth of plant leaf cells thus promoting earlier green-up (Kelsey et al., 2021). Due to larger leaf surface area, woody plants are more sensitive to increases in spring temperatures and radiation. Thus, encroached wetlands showed a higher negative response to spring warming compared to pure wetlands (Gu et al., 2022).

The impact of WPE on POS was primarily controlled by winter precipitation, summer temperature, and summer precipitation. Summer warming promotes photosynthesis by enhancing enzyme activity and cell growth, facilitating the arrival of the POS (Huang et al., 2019; Kobayashi et al., 2023). Conversely, increases in summer precipitation elevate soil moisture, leading to anaerobic respiration and accumulation of harmful substances, inhibiting plant growth (Sasidharan et al., 2018). Woody encroachment, with its high leaf surface area, absorbs more radiation and improved gas exchange efficiency, thereby reducing the impact of anaerobic respiration (Xie et al., 2007; Zhang et al., 2022). Consequently, WPE amplified the positive effects of summer warming and mitigated the negative effects of increased summer precipitation, ultimately resulting in earlier POS in encroached wetlands compared to pure wetlands.

The response of EOS to WPE was primarily controlled by summer temperature, summer precipitation and autumn temperature. The mechanism by which autumn temperatures regulated the effects of WPE on EOS was previously discussed, while the influence of summer temperatures and precipitation primarily stemmed from the following reasons. Summer warming intensifies evapotranspiration, resulting in increased soil moisture loss, which in turn worsens plant water stress and accelerates leaf withering and shedding (Jiao et al., 2019). Conversely, increased summer rainfall offsets these adverse effects, delaying EOS. Due to the ability of woody plants to access groundwater, encroached wetlands exhibited less sensitivity to the two climatic variables compared to pure wetlands (Huxman et al., 2005; Scott et al., 2014). Consequently, summer warming widened the EOS gap between encroached and pure wetlands, while increased summer rainfall had the opposite effect.

### 4.3 Limitations

Although our results provided a strong basis for evaluating the effects of WPE on phenology in boreal wetland ecosystems, there were several limitations in our study. First, the MCD12Q2 data had a spatial resolution of 500 m, which may affect the accuracy in detecting plant phenological events, particularly in highly heterogeneous wetland ecosystems (Piao et al., 2019). Thus, high-resolution satellite data and more accurate models are needed to further assess changes in vegetation phenology. Secondly, phenological changes could also be influenced by other factors that were not considered here, such as land use change, and droughts (Liu et al., 2016; Li et al., 2021; Pan et al., 2023). Therefore, future research should comprehensively consider the effects of these factors on phenological responses to WPE in boreal wetland ecosystems.

## 5 Conclusions

This study explored the regional phenological responses to WPE and the underlying mechanisms in boreal wetland ecosystems at middle-high latitudes. Our results revealed that WPE into boreal wetlands significantly advanced SOS and POS, but delayed EOS, thus extending GSL. Moreover, these effects of WPE on phenology increased over time. The climatic drivers not only directly regulated the effects of WPE on phenology but also indirect mediated it by regulating the degree of encroachment. These findings offer a new perspective on understanding the interaction between WPE and wetland ecosystems, highlighting the influence of WPE on biogeochemical cycles and climate feedback mechanisms.

## Data availability statement

The data analyzed in this study is subject to the following licenses/restrictions: Data will be made available on request. Requests to access these datasets should be directed to WW: wangwenj@iga.ac.cn.

## Author contributions

HS: Conceptualization, Data curation, Formal analysis, Investigation, Methodology, Software, Visualization, Writing – original draft, Writing – review & editing. WW: Conceptualization, Data curation, Funding acquisition, Methodology, Supervision, Writing – original draft, Writing – review & editing. ZL: Conceptualization, Supervision, Validation, Writing – original draft, Writing – review & editing. LW: Data curation, Writing – review & editing. SB: Investigation, Writing – review & editing. SB: Investigation, Writing – review & editing. YC: Investigation, Writing – review & editing.
