# Supplementary material for: Woody encroachment induced earlier and extended growing season in boreal wetland ecosystems
Source: Front Plant Sci. 2024 May 15;15:1413896. doi: 10.3389/fpls.2024.1413896 (PMC11133685; doi:10.3389/fpls.2024.1413896)
Supplement: Supplementary file 1 [file DataSheet_1.docx]

**Supplementary Information Appendix**

**Woody encroachment induced earlier and extended growing season in boreal wetland ecosystems**

Hongchao Sun^1,2^, Wen J. Wang^1,^*, Zhihua Liu^3^, Lei Wang^1^, Suri G. Bao^1,2^, Shengjie Ba^1,4^, Yu Cong^1^

^1.^ State Key Laboratory of Black Soils Conservation and Utilization, Northeast Institute of Geography and Agroecology, Chinese Academy of Sciences, Changchun, 130102, China

^2.^ University of Chinese Academy of Sciences, Beijing 100049, China

^3.^Institute of Applied Ecology, Chinese Academy of Sciences, Shenyang 110016, China

^4^ School of Geographical Sciences, Northeast Normal University, Changchun 130024, China

**Corresponding authors**: Wen J. Wang: wangwenj@iga.ac.cn.

**Text. S1. Method for quantifying the specific 100% WPE effects on wetland SOS, POS, EOS and GSL**

We employed a methodology from previous studies by Wang et al. (2018, 2021) to quantify the specific effects of 100% WPE on SOS, POS, EOS and GSL in wetlands (*θ*_SOS_, *θ*_POS_, *θ*_EOS_ and *θ*_GSL_). Conceptually, the SOS of a tree-encroached wetland (TEW) pixel (SOS_TEW_) were composed of the green-up date of wetlands (SOS_wetland_) and trees (SOS_tree_) within given TEW pixel (Figure S3, Equations 1).

| $\mathrm{SOS}_{\mathrm{TEW}}=$α × $\mathrm{SOS}_{\mathrm{tree}}$+ (1-α) × $\mathrm{SOS}_{\mathrm{wetland}}$ | (1) |
| --- | --- |

Where SOS_TEW_ was the actual SOS value of a given TEW pixel which was directly obtained from the MCD12Q2 phenology products; α was the fraction of trees in the given TEW pixel, indicating the degree of WPE; (1−α) referred to the fraction of wetland in the TEW pixel; SOS_tree_ represented the potential SOS of the given TEW pixel that was 100% encroached by trees (α=100%); SOS_wetland_ was the potential SOS of the given TEW pixel under pure cover of wetlands. Thus, mathematically, Equation 1 can be transformed to Equations 2–4.

| $\mathrm{SOS}_{\mathrm{TEW}}-\mathrm{SOS}_{\mathrm{wetland}}=\alpha\times(\mathrm{SOS}_{\mathrm{tree}}- \mathrm{SOS}_{\mathrm{wetland}})$ | (2) |
| --- | --- |

| $\frac{\mathrm{SOS}_{\mathrm{TEW}} - \mathrm{SOS}_{\mathrm{wetland}}}{\alpha}= \mathrm{SOS}_{\mathrm{tree}}- \mathrm{SOS}_{\mathrm{wetland}}$ | (3) |
| --- | --- |

We used the term *β*_SOS_ to denote the left-hand term of Equation 2, quantifying the relative effect on the SOS when the encroachment degree was given as α within the specified window (Equation 5). We also employed the term *θ*_SOS_ to denote the right-side term of Equation 3, quantifying the specific effects of 100% WPE on wetland SOS when the wetland pixel was 100% encroached by trees (Equation 5). We then estimated the regional *θ*_SOS_ as the slope of a linear regression between the left-side term of *β*_SOS_ and α in Equation 3, utilizing data from all the selected windows.

$\beta_{\mathrm{SOS}}=\mathrm{SOS}_{\mathrm{TEW}}-\mathrm{SOS}_{\mathrm{wetland}}$ (4)

$\theta_{\mathrm{SOS}}=\mathrm{SOS}_{\mathrm{tree}}-\mathrm{SOS}_{\mathrm{wetland}}$ (5)


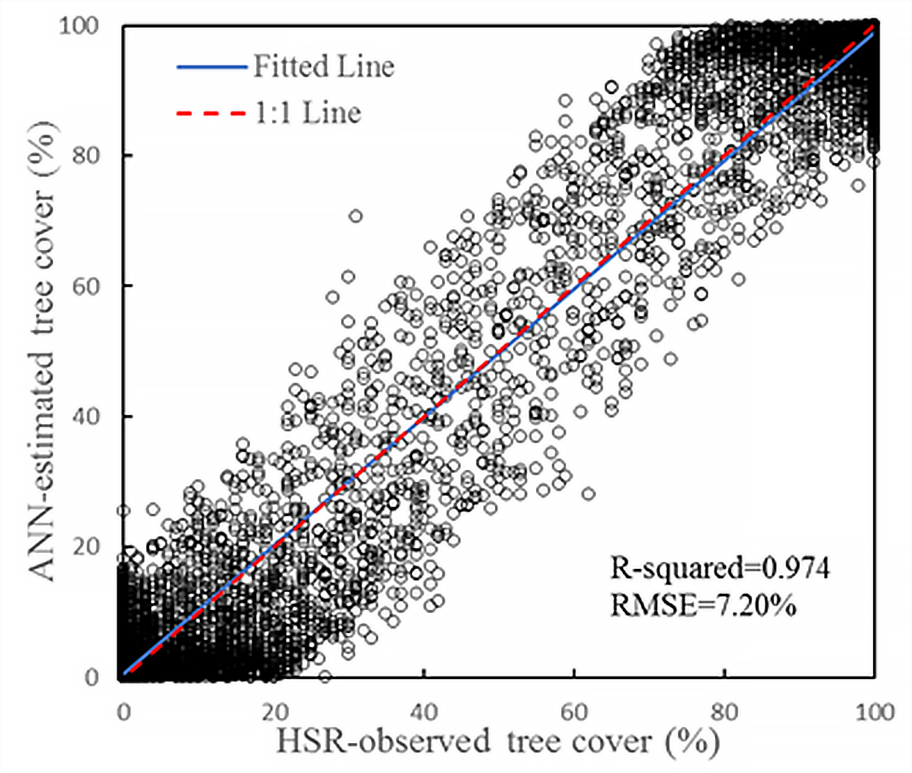


**Figure S1** Accuracy assessment of the estimated tree cover fractions against the observed fractional cover using ANN model.

**
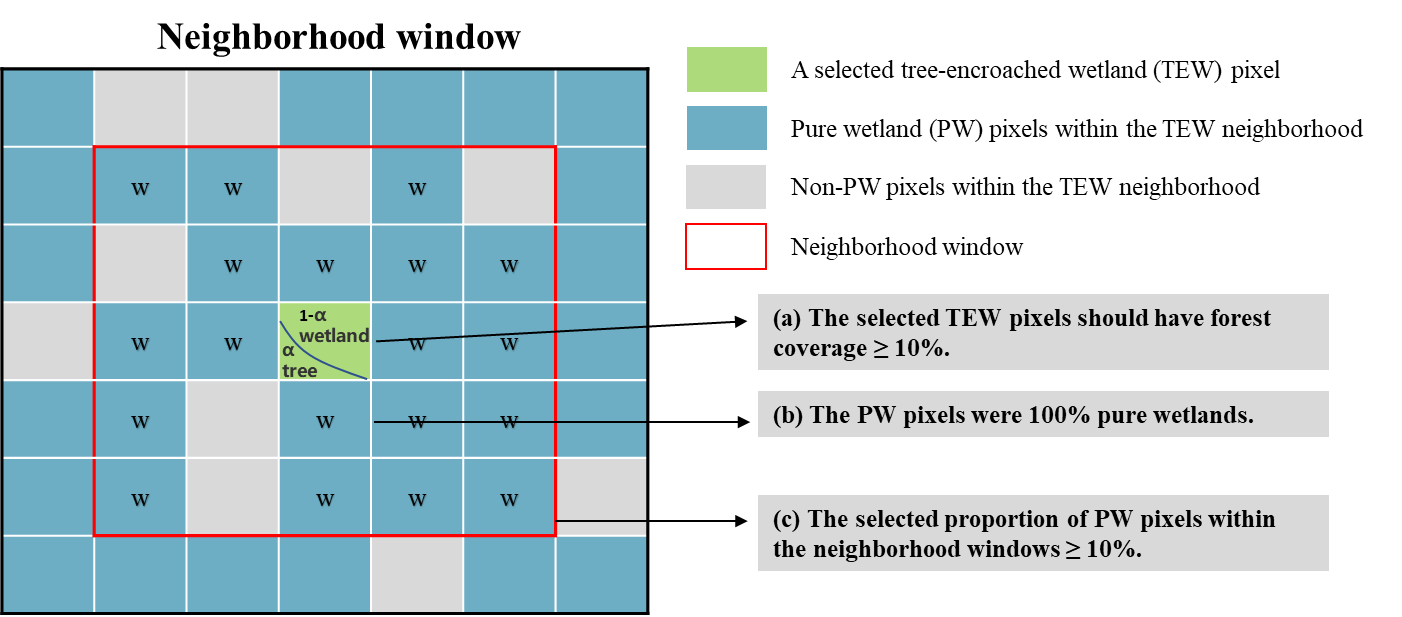
**

**Figure S2** A schematic illustration of the window searching strategy used in this study.


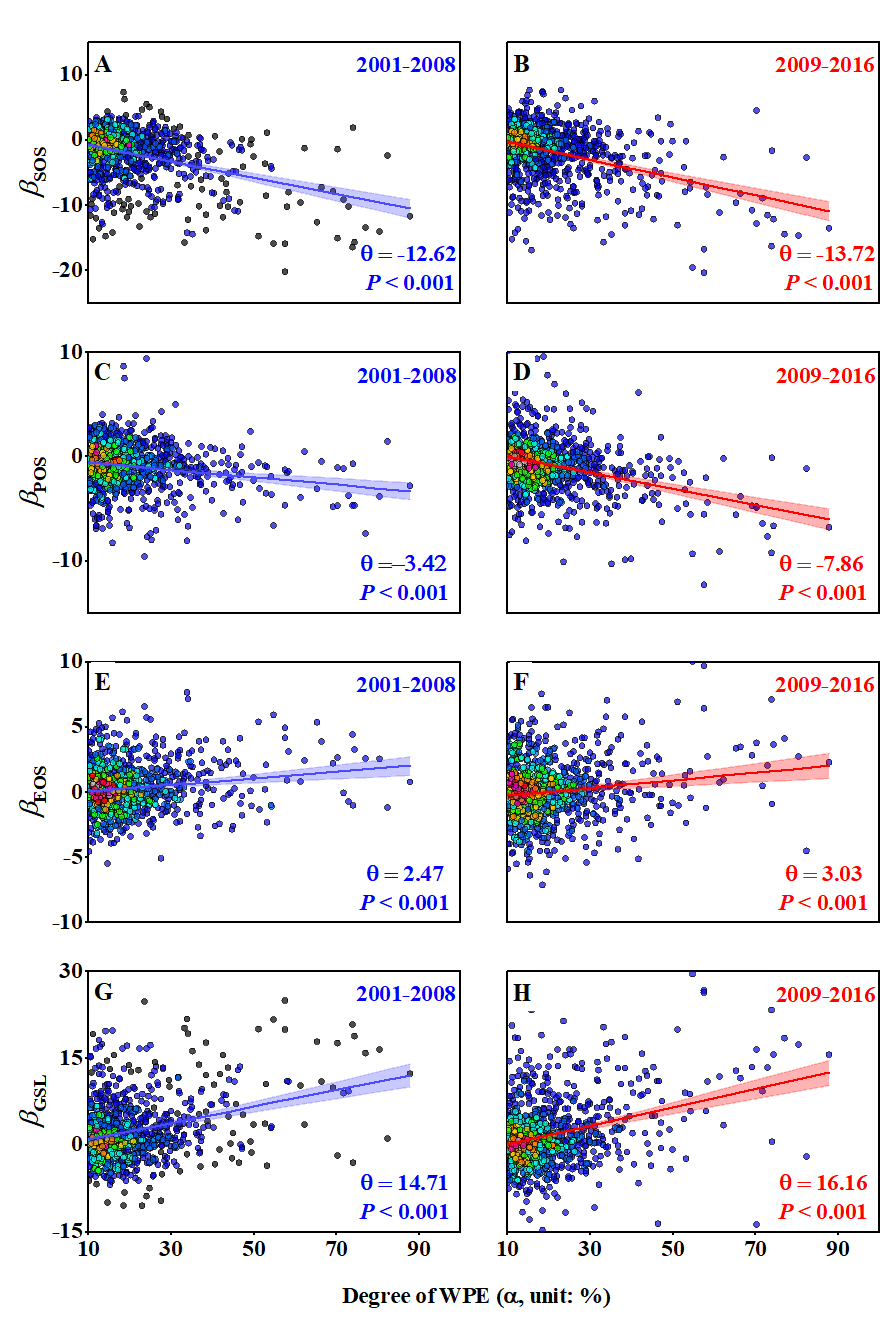


**Figure S3** The multi-year average effects of varying degrees (α) of WPE (*β*_SOS_, *β*_POS_, *β*_EOS_ and *β*_GSL_) and the specific effects of 100% WPE (*θ*_SOS_, *θ*_POS_, *θ*_EOS_ and *θ*_GSL_) on wetland SOS (A, B), POS (C, D), EOS (E, F) and GSL (G, H) based on the selected neighborhood windows of 21 × 21 pixels.


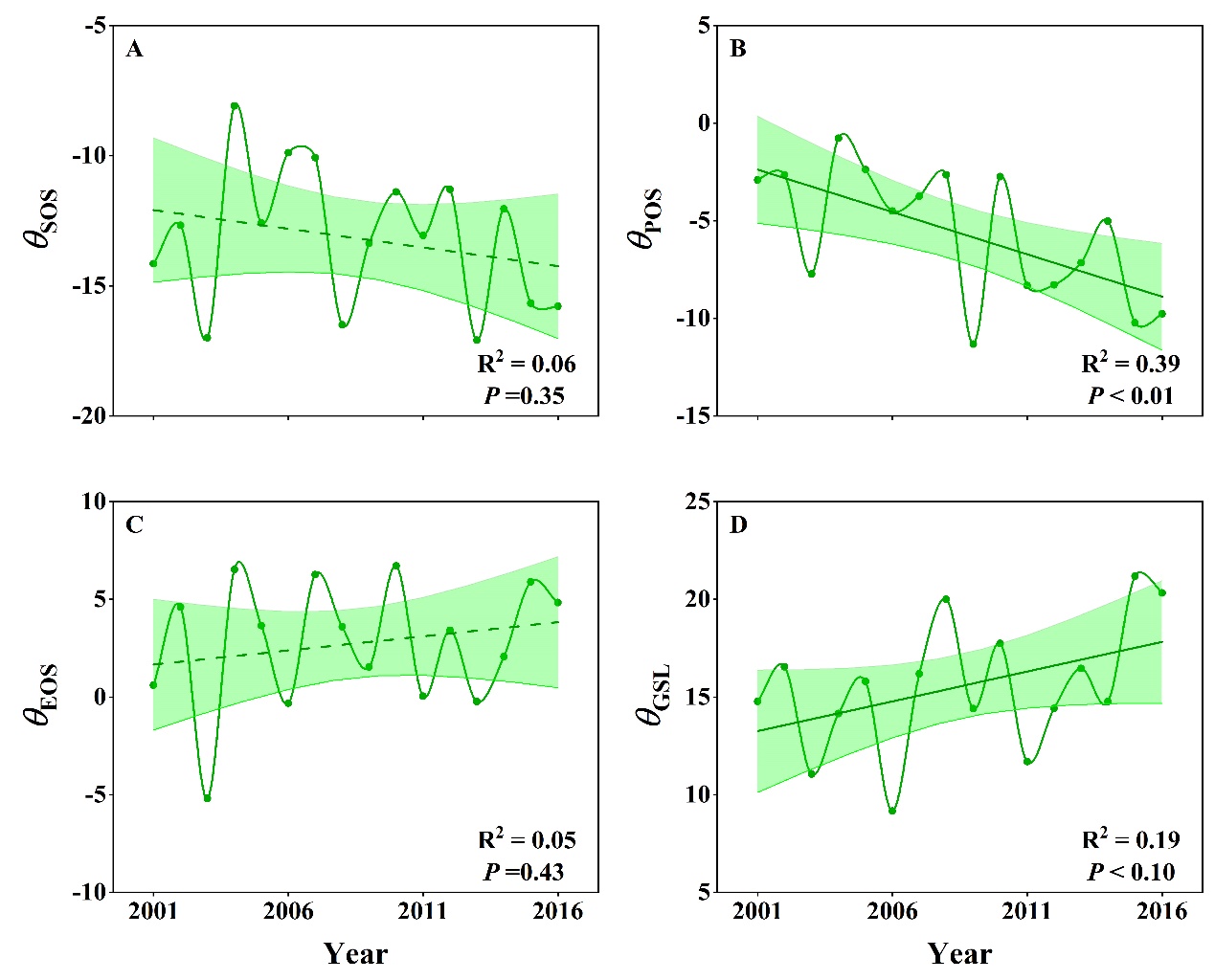


**Figure 4** Interannual dynamics (A, B, C and D) of the specific effects of 100% WPE on wetland SOS (*θ*_SOS_), POS (*θ*_POS_), EOS (*θ*_EOS_) and GSL (*θ*_GSL_) based on the selected neighborhood windows of 21 × 21 pixels.


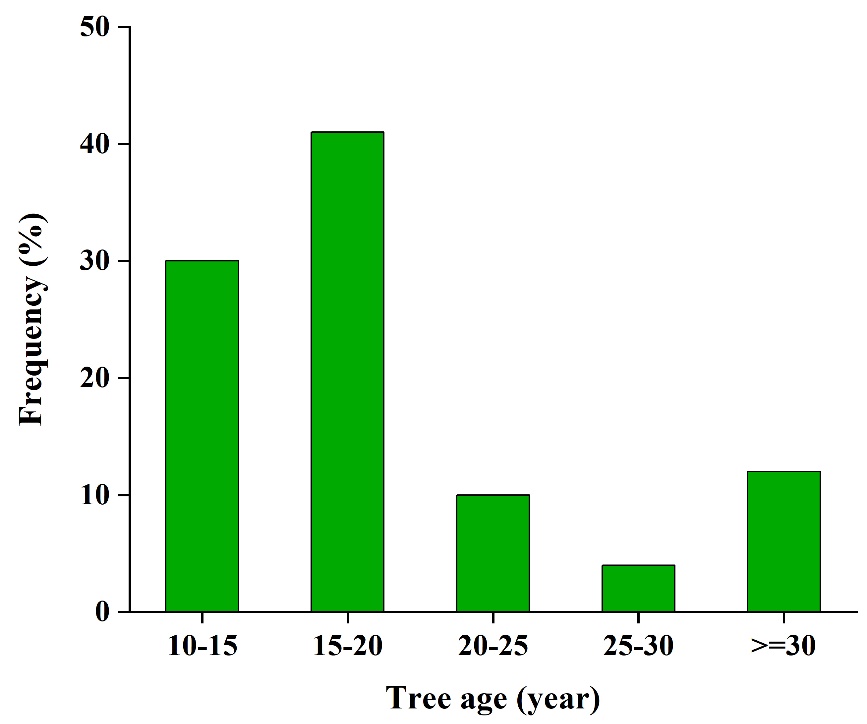


**Figure S5** Frequency distribution of age for the encroached tree samples in our study area.

**REFERENCES**

Wang, J., Xiao, X. M., Basara, J., Wu, X. C., Bajgain, R., Qin, Y. W., et al. (2021). Impacts of juniper woody plant encroachment into grasslands on local climate. Agric. For. Meteorol. 307, 108508. doi: 10.1016/j.agrformet.2021.108508

Wang, J., Xiao, X. M., Zhang, Y., Qin, Y. W., Doughty, R. B., Wu, X. C., et al. (2018). Enhanced gross primary production and evapotranspiration in juniper-encroached grasslands. Glob. Chang. Biol. 24(12), 5655-5667. doi:10.1111/gcb.14441
